# Breast cancer epidemiology and clinical outcomes in Moroccan women: a six-year retrospective study

**DOI:** 10.11604/pamj.2024.49.120.42588

**Published:** 2024-12-13

**Authors:** Fatiha Aboulhoda, Ouassima Erefai, Fadia Bejja, Abdelmajid Soulaymani, Abdelrhani Mokhtari, Hinde Hami

**Affiliations:** 1 Laboratory of Biology and Health, Faculty of Science, Ibn Tofail University, Kenitra, Morocco,; 2 Higher Institute of Nursing Professions and Health Techniques, Rabat, Morocco

**Keywords:** Breast cancer, epidemiology, women’s health, diagnosis delay, clinical features

## Abstract

**Introduction:**

breast cancer is the most commonly diagnosed cancer among women in Morocco, with 11,747 new cases reported in 2020. This study aimed to provide a comprehensive overview of the epidemiological and clinical aspects of breast cancer in Moroccan women.

**Methods:**

this retrospective study included all female breast cancer patients diagnosed at the Reference Center for Reproductive Health in Kenitra from 2013 to 2018. A detailed analysis of the patients´ medical records was conducted in this study.

**Results:**

the study included 973 female participants. The mean age of the patients was 51.1 ± 12.3 years (range, 18-95 years). Notably, 65.1% of the participants were from urban areas. The median diagnosis delay was 8 days. Over half (53.3%) of the women were postmenopausal, with a mean age at menopause of 50.3 ± 4 years. The mean age at first pregnancy was 23.9 ± 6.8 years, ranging from 12 to 47 years. Fifty-four percent of the women used hormonal contraceptives, primarily oral pills. During clinical examination, breast nodules were the most prevalent presenting sign in the sampled women, with nodules typically found to be fixed in 89% of cases in the right breast and 91% in the left breast.

**Conclusion:**

the study revealed significant concerns regarding women diagnosed at the Reference Center for Reproductive Health in Kenitra, such as prolonged diagnosis delays, advanced age at menopause, and a considerable prevalence of nodules and palpable axillary adenopathy.

## Introduction

Breast cancer is the most common type of cancer affecting women worldwide, in both developed and developing countries. Currently, there are approximately 2.26 million reported cases of breast cancer worldwide [1]. A concerning statistic indicates that one out of every twelve women will experience this type of cancer in their lifetime [1]. Breast cancer is a leading cause of death among women, contributing to approximately 685,000 fatalities [2]. This issue assumes even greater significance in low- and middle-income countries. In Africa, the breast cancer death rate is the highest [1].

In Morocco, breast cancer has rapidly become a significant health concern because of factors such as higher life expectancy, urbanization, and the adoption of Western lifestyles [3]. Breast cancer accounts for a substantial 38% of all cancer cases in women and 22.5% of all cancers in both sexes [4]. The Greater Casablanca Region alone had an estimated incidence rate of 51.2 cases per 100,000 women between 2013 and 2017 [4]. Moreover, breast cancer is the leading cause of female cancer-related deaths in Morocco, causing 3,518 fatalities, which accounts for 25% of all female cancer-related fatalities [1].

Although breast cancer can affect women of any age, older women are more prone to being diagnosed with the disease [4]. Clinical indications for breast cancer include lumps, nodules, discharge, and changes in the skin's appearance. Preventing a late-stage diagnosis is crucial through early detection and diagnosis [5].

Numerous epidemiological studies have examined breast cancer in university hospitals. However, this study is the first to present findings from the Reference Center for Reproductive Health in Kenitra. The objective of this study was to describe the epidemiological features, reproductive, hormonal, and clinical characteristics of Moroccan women diagnosed with breast cancer.

## Methods

**Study design:** this retrospective study focused on women diagnosed with breast cancer between January 1^st^, 2013, and December 31^st^, 2018, in Kenitra, Morocco.

**Study setting and data collection:** this study was conducted at the Reference Center for Reproductive Health (RCRH) in Kenitra. This facility is devoted to providing assistance to primary health care facilities (PHCFs) and private practices. RCRH provides outstanding diagnostic care tailored for women referred from both urban and rural health centers, with a specialization in reproductive health. The study included women not only from the Kenitra province but also from Sidi Slimane (located 70 km away from Kenitra) and Sidi Kacem (situated 90 km away from Kenitra).

The data were extracted from the medical records of the patients, including also the radiological and histological reports, for all women who had been diagnosed and confirmed with breast cancer. It is acknowledged that the variables available are limited due to the monocentric nature of the data collection. It is considered that the data provide valuable information for the field.

**Study population:** the study included all women who underwent breast anomaly screening at primary healthcare institutions or private practices in Kenitra, Sidi Slimane, and Sidi Kacem. Only those diagnosed with breast cancer through radiological means (echo-mammography) and histological examinations at the RCRH were eligible for inclusion in this study.

**Study variables:** data on participant characteristics were collected for this study, starting with demographic variables such as age, marital status, place of residence (rural or urban), geographic origin, and profession. In addition, diagnostic-related factors were examined, such as the time to diagnosis (the duration between the reception date at RCRH and diagnosis). The study assessed obstetric and reproductive variables, such as parity (the number of pregnancies carried to term), age at first pregnancy, and use of hormonal contraception, as well as physical characteristics, including breast contour and skin surface features. The clinical variables evaluated included nodules within the breast, axillary adenopathy, affected breast, and the presence of any breast discharge. This study also included menstrual cycle characteristics as part of the comprehensive data.

**Statistical analysis:** data were analyzed using IBM SPSS Statistics 21.0 software, with methods chosen for their relevance to the specific aims of our study on breast cancer in Moroccan women. Descriptive statistics were applied to the demographic and diagnostic data. Frequencies were used for categorical variables, while means with standard deviations were used for quantitative variables such as age, parity, duration of hormonal contraception, and annual case numbers. The median and interquartile range were used to characterize the time to diagnosis because of its non-normal distribution. This provides a detailed perspective on potential diagnostic delays and ensures that our analysis directly correlates with the intricate epidemiological and clinical patterns we aim to uncover.

## Results

**Figure 1:**
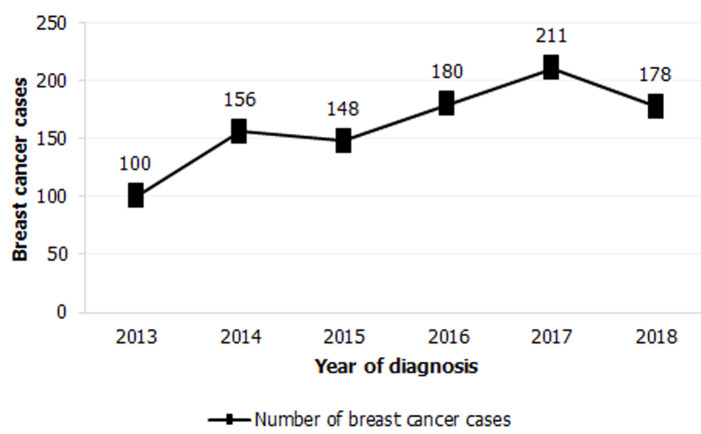
annual evolution of breast cancer cases at the Reference Center for Reproductive Health, Kenitra

**Geographic distribution and diagnosis delays:** during the 6-year study, 973> women were diagnosed with breast cancer (Figure 1). On average, 162 ± 37.6 new cases were reported each year. In terms of geographical distribution, 67.2% (n = 650) of the participants were from Kenitra, 17.2% (n = 166) of them were from Sidi Slimane, and 15.6% (n = 151) of the participants hailed from Sidi Kacem.

The median diagnosis delay was 8 days (Q1-Q3: 6-14 days), calculated as the period between the reception date at RCRH and the diagnosis date. Patients from different regions experienced only slightly varying diagnostic delays. Patients from Sidi Slimane and Sidi Kacem had a median delay of 7 days (Q1-Q3: 5-13 days), whereas patients from Kenitra experienced a median delay of 8 days (Q1-Q3: 6-16 days).

**Characteristics of study participants:** the mean age of breast cancer diagnosis was 51.1 ± 12.3 years, ranging from 18 to 95 years. Most cases (76%) were detected in the age group of 40-69 years, which coincides with Morocco´s screening program for early detection of breast and cervical cancer. Furthermore, 15.9% of cases involved patients under the age of 40, and 8.1% affected women aged 70 years or older. Regarding marital status, most patients (66.4%) were married. Notably, 65.1% of the patients lived in urban areas (Table 1).

**Table 1:** sociodemographic characteristics of study participants

Characteristics	Number of new cases	Percentage (%)
**Age group (years) (n = 973)**		
< 30	24	2.5
30-40	163	16.8
41-50	310	31.9
51-60	276	28.4
> 60	200	20.6
**Marital status (n = 896)**		
Married	595	66.4
Unmarried	106	11.8
Divorced	66	7.4
Widowed	129	14.4
**Place of residence (n = 896)**		
Rural	337	34.9
Urban	630	65.1
**Origin (n = 967)**		
Kenitra	650	67.2
Sidi Slimane	166	17.2
Sidi Kacem	151	15.6
**Profession (n = 841)**		
Not employed	786	93.5
Self-employed	21	2.5
Civil servant	26	3.1
Other	8	1.0

**Reproductive and hormonal factors:** postmenopausal women constituted a considerable proportion (53.3%) of breast cancer cases among diagnosed women (Table 2). In contrast, pregnant and lactating women accounted for only a small portion (2%) of the cases, with 9 and 12 pregnancies identified, respectively. The cohort´s mean age at menopause was determined to be 50.3 ± 4 years. Examining pregnancy history, most women (81%) had experienced pregnancies, with a mean of 4.4 pregnancies and a mean parity of 3.9 children per woman. The women in the study population experienced their first pregnancy at a mean age of 23.9 ± 6.8 years, ranging from 12 to 47 years. Furthermore, 84.5% of women became pregnant before reaching the age of 30. When analyzing the data collected from female participants, the study found that 26.1% (n = 186) had undergone fewer than two pregnancies, 67.2% (n = 480) had between two and eight pregnancies, and 6.7% (n = 48) had experienced more than eight pregnancies. More than half of the patients (53.9%) used contraceptive pills for a mean duration of 8.9 years. Hormone replacement therapy was only used by 1.3% (n = 10) of the patients, with a mean duration of 15.4 years.

**Table 2:** reproductive and health characteristics of study participants

Characteristics	Number of new cases	Percentage (%)
**Menopausal status (n = 806)**		
Menopausal	430	53.3
Non-menopausal	376	46.7
**Age at menopause (n = 294)**		
< 50 years	149	50.7
50 + years	147	49.3
**Age at first pregnancy (n = 652)**		
< 30 years	551	84.5
30 + years	101	15.5
**Menstrual cycle (n = 355)**		
Irregular cycle	85	23.9
Regular cycle	270	76.1

**Clinical features:** the clinical evaluation of women showed that the most common early indicator was the presence of a breast lump. In most cases, these lumps were stationary, with 89% detected in the right breast and 91% in the left breast. Palpable axillary adenopathy was noted on the right side in 527 women and on the left side in 264 women. Irregular breast contour was primarily indicated by retraction, with almost equal occurrences in both the right and left breasts, at 64% and 66%, respectively. Frequently, skin surface anomalies were noted, especially the “orange peel” texture, observed in 38% of right breast cases and 41% of left breast cases. Redness was the second most prevalent skin surface abnormality in 20% of right breast cases and 15% of left breast cases. Regarding the discharge, hemorrhagic discharge was experienced by only 15 women on the right side, whereas there were five on the left side.

## Discussion

Despite significant advances in cancer prevention and treatment, the incidence of breast cancer continues to increase annually. Breast cancer continues to be one of the most common cancer types worldwide and is a major contributor to cancer-related deaths [3]. In Morocco, breast cancer is particularly prevalent, representing 22.5% of all cancer cases documented between 2013 and 2017 [4]. In our study, patients were diagnosed with breast cancer at a mean age of 51.1 ± 12.3 years. Interestingly, 15.9% of cases occurred in women under the age of 40 years, whereas 8.1% affected women aged 70 years or older. These results are consistent with those of a recent study conducted in Morocco [6]. In contrast, the mean ages for breast cancer diagnosis in French women are relatively older, around 59 and 67 years [7,8]. However, some African countries, including Niger (44.1 years), Libya (45.5 years), and Cameroon (46.6 years) have reported lower ages at breast cancer diagnosis [9-11]. The variation in the diagnosis age across countries might arise from earlier life exposures to different risk factors.

A large proportion of our patients (65.1%, n = 630) originated from urban areas, likely because of the availability of medical services in metropolitan regions. In addition, 93.5% of the women surveyed identified themselves as housewives, which could impact their healthcare-seeking behavior.

An early diagnosis of breast cancer is crucial for the effective management of the disease and for improving patient outcomes. However, several factors, including socio-economic, psychological, geographical, organizational, and institutional aspects, affected the time to diagnosis in our study cohort. The mean diagnosis time was calculated to be 25 ± 2.4 days, which is longer than the shorter durations reported in other nations such as Tunisia (13 days) and France (17.7 days) [12]. Furthermore, a study conducted at a tertiary-level facility in Morocco reported a longer duration of diagnosis (33.5 days) [6]. The extended delays in diagnosing breast cancer cases in Morocco highlight the need for interventions aimed at mitigating them, thereby promoting improved detection and treatment.

When talking about risk factors for breast cancer, the age at which a woman has her first pregnancy plays an important role in its development. Delayed first pregnancies (age 35 or older compared to age 20 or younger) are associated with a 1.5-fold increased risk of developing breast cancer [13]. However, this risk is reduced if the first pregnancy occurs before the age of 30 [14]. In addition, the risk of breast cancer decreases with pregnancies at younger ages [15], whereas delayed pregnancies tend to correspond with more aggressive forms of breast cancer [16,17].

In our study population, the mean age for a woman´s first pregnancy before 30 years of age was 21.7 years, which agrees with findings from a previous investigation conducted in Morocco [18]. Additionally, studies indicate that full-term pregnancies confer a protective effect against breast cancer, reducing the risk by 25% among women who have had at least one full-term pregnancy [19]. In contrast, delayed menopause increases vulnerability to breast cancer, primarily due to prolonged exposure to estrogen [14,20]. Advancing age at menopause is a risk factor, with each year beyond 50 years correlated with a 3% increased risk [14,21]. Sancho-Garnier *et al*. emphasized that the elevated risk associated with late menopause becomes more pronounced after the age of 55 [22]. More than half (53.6%) of the women in our sample had already reached menopause, with 50% of them being above 50 years of age. As per a previous study, prolonged use of oral contraceptives and age have been linked to an increased risk of breast cancer [23]. In our study, a significant number of women (53.9%, n = 426) reported using hormonal contraceptives (the pill), which is notably higher than that reported in a previous study conducted in Morocco [24].

Research on clinical indicators that illuminate their role in breast cancer is limited [25]. On occasion, these clinical signs may not be immediately noticeable, and the lesion is only discovered incidentally during mammographic or ultrasound assessments [26]. A study conducted in Cameroon revealed that clinical examinations may not always indicate breast cancer or infection signs [27]. Moreover, according to another study, small subclinical cancers may not always be detectable through clinical examinations [15]. Nevertheless, specific studies have highlighted the importance of clinical examinations in the diagnosis of breast cancer. A descriptive study found that among the cases examined, 92.4% had masses, 72.9% had skin alterations, and 83.3% had discharge [28]. In Tunisia, 80.3% of cases showed a palpable mass, 24.4% experienced pain, 3.8% reported discharge, and 1.9% had inflammation [12]. In our patient cohort, clinical examination was the primary screening method for detecting at least one clinical anomaly in one or both breasts.

**Limitations:** this study provides significant insights into the epidemiology and clinical characteristics of breast cancer among Moroccan women. However, its retrospective design limits its findings due to potential incomplete data, which may lead to the underestimation of certain variables. Furthermore, the absence of specific timelines from initial screening to diagnosis limits our complete understanding of the factors contributing to diagnostic delays, which could affect the interpretation of their impact on clinical outcomes and disease progression.

Additionally, conducting research solely within a diagnostic center has restricted the longitudinal perspective of disease trajectories post-diagnosis, including treatment responses and long-term patient outcomes. Furthermore, the absence of staging information limits our ability to fully stratify prognoses and customize treatment interventions.

However, our study contributes critical epidemiological and clinical insights, significantly enriching the discourse on breast cancer within the Moroccan context. The findings have significant implications for policy and practice, particularly in advocating for integrated screening programs and the adoption of multimodal diagnostic approaches. Recognizing these limitations underscores the imperative for further research. Subsequent studies should expand upon these initial findings by applying prospective methodologies that cover the entire spectrum of breast cancer management, from early detection to survivorship or end-of-life care.

**Future research directions:** research in breast oncology is pivotal for enhancing our understanding of the disease and for developing superior prevention and treatment modalities. This study highlights significant diagnostic delays in the Moroccan context, necessitating further investigation into the underlying factors. Future research should rigorously investigate the time interval between symptom onset and initial screening and between screening and definitive diagnosis. Delving into treatment accessibility and its impact on survival rates, therapeutic outcomes, and prognostic determinants will further elucidate breast cancer management in Morocco. Such inquiries are essential for improving our understanding and enhancing patient care and survival prospects.

## Conclusion

This study highlights concerning aspects related to women´s diagnoses at the RCRH in Kenitra. These include significant diagnostic delays, advanced age at menopause, presence of nodules, and palpable axillary adenopathy. The identification of advanced breast cancer cases despite the implementation of the breast and cervical cancer early detection program in Kenitra since 2013 has raised questions about the efficacy of relying solely on clinical screening. Therefore, it is essential to consider the integration of radiological screening as a complementary or alternative approach to clinical methods to significantly improve early detection rates.

### What is known about this topic

- Breast cancer is the most prevalent malignancy among women worldwide, with significant mortality rates, particularly in low- and middle-income countries;
- In Morocco, breast cancer is the leading cause of cancer-related mortality in women;
- the increasing incidence of breast cancer has been linked to demographic transitions, such as increased life expectancy, urbanization, and lifestyle changes.

### What this study adds

- This study provides a comprehensive epidemiological and clinical analysis of breast cancer among women in Kenitra, Morocco;
- the study highlights prolonged diagnostic delays and a notably high prevalence of advanced-stage cases, which were previously undocumented;
- The findings illuminate critical facets of breast cancer diagnosis in Moroccan women, including age at onset, reproductive health factors, and specific clinical indicators; this provides a distinctive insight into breast cancer patterns in the region.
